# 1-Formyl-*r*-2,*c*-6-bis­(4-methoxy­phen­yl)-*t*-3-methyl­piperidin-4-one

**DOI:** 10.1107/S1600536809042457

**Published:** 2009-10-23

**Authors:** P. Gayathri, P. Sakthivel, S. Ponnuswamy, A. Thiruvalluvar, R. J. Butcher

**Affiliations:** a PG Research Department of Physics, Rajah Serfoji Government College (Autonomous), Thanjavur 613 005, Tamilnadu, India; b Department of Chemistry, Government Arts College (Autonomous), Coimbatore 641 018, Tamilnadu, India; c Department of Chemistry, Howard University, 525 College Street NW, Washington, DC 20059, USA

## Abstract

The asymmetric unit of the title compound, C_21_H_23_NO_4_, contains two crystallographically independent mol­ecules *A* and *B*. In both mol­ecules, the piperidine-4-one rings adopt a distorted twist-boat conformation. The formyl group at position 1, the methoxy­phenyl ring at position 2 and the methyl group at position 3 are attached equatorially. The meth­oxy phenyl ring at position 6 has an axial orientation. The dihedral angle between the two benzene rings is 55.27 (8)° in mol­ecule *A*, and 55.29 (8)° in mol­ecule *B*. In the crystal, the mol­ecules are linked by weak C—H⋯O inter­molecular hydrogen-bond inter­actions. In addition, weak C—H⋯π inter­molecular inter­actions involving the benzene rings at positions 6 and 2 of mol­ecule *B* are also found in the crystal structure.

## Related literature

For the biological activity of piperidones, see: Aridoss *et al.* (2008 ▶). For anti­neoplastic agents, see: Pati *et al.* (2008 ▶). For the stereochemistry of piperidine-4-one, see: Ponnuswamy *et al.* (2002 ▶); Venkatraj *et al.* (2008 ▶).
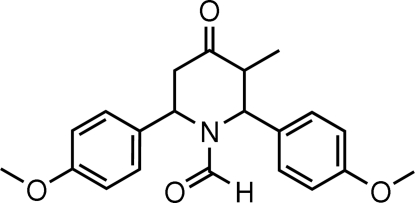


## Experimental

### 

#### Crystal data

C_21_H_23_NO_4_
*M* *_r_* = 353.40
Triclinic, 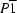

*a* = 11.5409 (4) Å
*b* = 12.4972 (5) Å
*c* = 14.7816 (6) Å
α = 67.878 (4)°
β = 74.719 (3)°
γ = 67.123 (4)°
*V* = 1802.42 (14) Å^3^
*Z* = 4
Cu *K*α radiation
μ = 0.73 mm^−1^
*T* = 110 K
0.55 × 0.45 × 0.18 mm

#### Data collection

Oxford Diffraction Xcalibur Ruby Gemini diffractometer
Absorption correction: multi-scan (CrysAlis Pro; Oxford Diffraction, 2009 ▶) *T*_min_ = 0.484, *T*_max_ = 1.000
13170 measured reflections
7096 independent reflections
6709 reflections with *I* > 2σ(*I*)
*R*_int_ = 0.020

#### Refinement

*R*[*F* ^2^ > 2σ(*F* ^2^)] = 0.046
*wR*(*F* ^2^) = 0.127
*S* = 1.06
7096 reflections
477 parameters
H atoms treated by a mixture of independent and constrained refinement
Δρ_max_ = 0.69 e Å^−3^
Δρ_min_ = −0.24 e Å^−3^

### 

Data collection: *CrysAlis Pro* (Oxford Diffraction, 2009 ▶); cell refinement: *CrysAlis Pro*; data reduction: *CrysAlis Pro*; program(s) used to solve structure: *SHELXS97* (Sheldrick, 2008 ▶); program(s) used to refine structure: *SHELXL97* (Sheldrick, 2008 ▶); molecular graphics: *ORTEP-3* (Farrugia, 1997 ▶); software used to prepare material for publication: *PLATON* (Spek, 2009 ▶).

## Supplementary Material

Crystal structure: contains datablocks global, I. DOI: 10.1107/S1600536809042457/jj2013sup1.cif

Structure factors: contains datablocks I. DOI: 10.1107/S1600536809042457/jj2013Isup2.hkl

Additional supplementary materials:  crystallographic information; 3D view; checkCIF report

## Figures and Tables

**Table 1:** Hydrogen-bond geometry (Å, °)

*D*—H⋯*A*	*D*—H	H⋯*A*	*D*⋯*A*	*D*—H⋯*A*
C5*A*—H5*B*⋯O16*B*^i^	0.99	2.47	3.351 (2)	148
C5*B*—H5*C*⋯O11*A*^ii^	0.99	2.34	3.222 (2)	148
C12*B*—H12*D*⋯O11*A*^iii^	0.98	2.47	3.427 (2)	167
C16*A*—H16*A*⋯O12*B*^iv^	0.98	2.51	3.468 (2)	167
C16*B*—H16*D*⋯O16*A*^v^	0.98	2.47	3.334 (2)	147
C25*A*—H25*A*⋯O4*B*^vi^	0.95	2.43	3.266 (2)	147
C2*A*—H2*A*⋯*Cg*1^vii^	1.00	2.71	3.714 (2)	178
C12*A*—H12*B*⋯*Cg*2^viii^	0.98	2.92	3.846 (2)	158

Symmetry codes: (i) 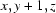; (ii) 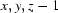; (iii) 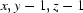; (iv) 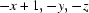; (v) 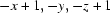; (vi) 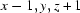; (vii) 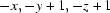; (viii) 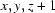. *Cg*1 and *Cg*2 are the centroids of the C61*B*–C66*B* and C21*B*–C26*B* rings, respectively.

**Fig. 1.**
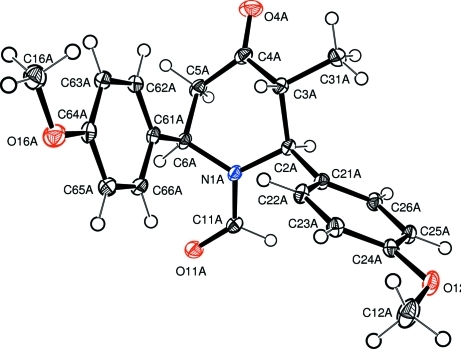
The molecular structure of independent molecule A, showing the atom-numbering scheme and displacement ellipsoids drawn at the 40% probability level(arbitary spheres for H atoms).

**Fig. 2.**
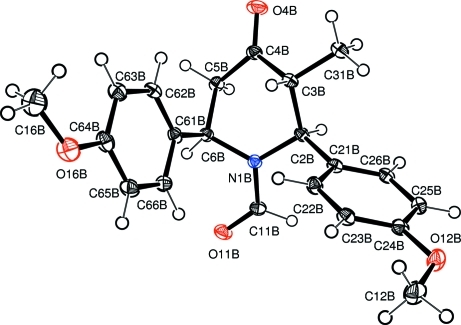
The molecular structure of independent molecule B, showing the atom-numbering scheme and displacement ellipsoids drawn at the 40% probability level(arbitary spheres for H atoms).

**Fig. 3.**
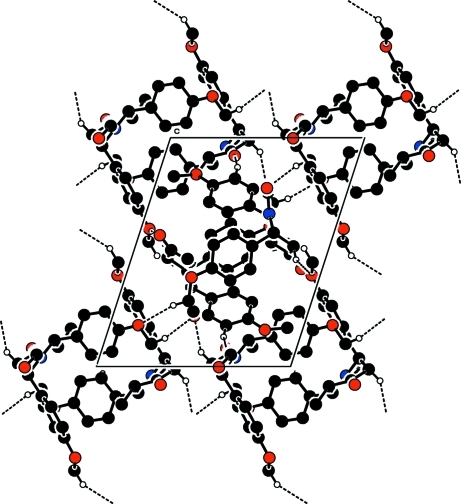
The packing of the title compound, viewed down the a axis. Dashed lines indicate weak C–H···O hydrogen bond intermolecular interactions. H atoms not involved in hydrogen bonding have been omitted.

## supplementary crystallographic information

### Comment

Piperidin-4-ones and their derivatives show a broad spectrum of biological activities which includes antimicrobial, antiviral, anti tuberculosis and anticancer activities and could also be used against multidrug resistant organisms (Aridoss *et al.*, 2008).  2,6-Diarylpiperidin-4-one based chemical entities act as antineoplastic agents (Pati *et al.*, 2008). Recent efforts devoted to 2,6-diarylpiperidin-4-one based chemical entities and establishing their stereochemistry (Ponnuswamy *et al.*, 2002), (Venkatraj *et al.*, 2008) are significant because the pharmacological effects of potential new drugs depends on the stereochemistry and ring conformations of these compounds.

The asymmetric unit of the title compound, C_21_H_23_NO_4_, contains two crystallographically independent molecules A and B (Fig. 1, Fig. 2). In both molecules, the piperidine ring adopts a distorted twist boat conformation. The formyl group at 1, the methoxy phenyl ring at 2 and the methyl group at 3 are attached equatorially.  The methoxy phenyl ring at 6 has an axial orientation. The dihedral angle between the two benzene rings is 55.27 (8)° in molecule A; and 55.29 (8)° in molecule B. Compound(I) is chiral: in the arbitrarily chosen asymmetric molecules, C2A(C2B), C3A(C3B) and C6A(C6B) have S, R and R conformations respectively. The molecules are linked by weak C–H···O intermolecular hydrogen bond interactions (Fig. 3; Table 1). In addition, weak C2A-H2A···π and C12A-H12B···π intermolecular interactions involving the benzene rings at positions 6(C61B-C66B) and 2(C21B-C26B) of molecule B are also found in the crystal structure.

### Experimental

The ice-cold solution of acetic-formic anhydride was prepared from acetic anhydride (10 ml) and 85% formic acid (5 ml) and was added slowly to a cold solution of r-2,c-6-bis(4-methoxyphenyl)-t-3-methylpiperidin-4-one (1.625 g, 0.005 mol) in benzene (30 ml). The reaction mixture was stirred at room temperature for 5 h. The organic layer was separated, dried over anhydrous Na_2_SO_4_ and concentrated. The resulting mass was purified by crystallization from benzene-petroleum ether (333–335 K) in the ratio 1:1. Yield obtained was 1.13 g (64%).

### Refinement

Atoms H11A at C11A and H11B at C11B were located in a difference Fourier map and refined isotropically. Remaining H atoms were positioned geometrically and allowed to ride on their parent atoms, with C—H = 0.95, 0.98, 0.99 and 1.00 Å for C*sp*^2^, methyl, methylene and methine C, respectively; *U*_iso_(H) = k*U*_eq_(C), where k = 1.5 for methyl and 1.2 for all other H atoms. The maximum residual electron density peak 0.685 e Å^-3^ is located 2.23Å from H5A.

### Crystal data

**Table:**

C_21_H_23_NO_4_	*Z* = 4
*M**_r_* = 353.40	*F*(000) = 752
Triclinic, *P*1	*D*_x_ = 1.302 Mg m^−^^3^
Hall symbol: -P 1	Melting point: 382(1) K
*a* = 11.5409 (4) Å	Cu *K*α radiation, λ = 1.54184 Å
*b* = 12.4972 (5) Å	Cell parameters from 11847 reflections
*c* = 14.7816 (6) Å	θ = 4.7–74.0°
α = 67.878 (4)°	µ = 0.73 mm^−^^1^
β = 74.719 (3)°	*T* = 110 K
γ = 67.123 (4)°	Plate, colourless
*V* = 1802.42 (14) Å^3^	0.55 × 0.45 × 0.18 mm

### Data collection

**Table:**

Oxford Diffraction Xcalibur Ruby Gemini diffractometer	7096 independent reflections
Radiation source: Enhance (Cu) X-ray Source	6709 reflections with *I* > 2σ(*I*)
graphite	*R*_int_ = 0.020
Detector resolution: 10.5081 pixels mm^-1^	θ_max_ = 74.2°, θ_min_ = 4.7°
ω scans	*h* = −14→13
Absorption correction: multi-scan (*CrysAlis PRO*; Oxford Diffraction, 2009)	*k* = −15→11
*T*_min_ = 0.484, *T*_max_ = 1.000	*l* = −18→17
13170 measured reflections	

### Refinement

**Table:**

Refinement on *F*^2^	Primary atom site location: structure-invariant direct methods
Least-squares matrix: full	Secondary atom site location: difference Fourier map
*R*[*F*^2^ > 2σ(*F*^2^)] = 0.046	Hydrogen site location: inferred from neighbouring sites
*wR*(*F*^2^) = 0.127	H atoms treated by a mixture of independent and constrained refinement
*S* = 1.06	*w* = 1/[σ^2^(*F*_o_^2^) + (0.0704*P*)^2^ + 1.0073*P*] where *P* = (*F*_o_^2^ + 2*F*_c_^2^)/3
7096 reflections	(Δ/σ)_max_ = 0.001
477 parameters	Δρ_max_ = 0.69 e Å^−^^3^
0 restraints	Δρ_min_ = −0.24 e Å^−^^3^

### Special details

**Table:**

Geometry. Bond distances, angles *etc*. have been calculated using the rounded fractional coordinates. All su's are estimated from the variances of the (full) variance-covariance matrix. The cell e.s.d.'s are taken into account in the estimation of distances, angles and torsion angles
Refinement. Refinement of *F*^2^ against ALL reflections. The weighted *R*-factor *wR* and goodness of fit *S* are based on *F*^2^, conventional *R*-factors *R* are based on *F*, with *F* set to zero for negative *F*^2^. The threshold expression of *F*^2^ > 2σ(*F*^2^) is used only for calculating *R*-factors(gt) *etc*. and is not relevant to the choice of reflections for refinement. *R*-factors based on *F*^2^ are statistically about twice as large as those based on *F*, and *R*- factors based on ALL data will be even larger.

### Fractional atomic coordinates and isotropic or equivalent isotropic displacement parameters (Å^2^)

**Table:**

	*x*	*y*	*z*	*U*_iso_*/*U*_eq_	
O4A	−0.07324 (12)	0.89236 (11)	0.42627 (9)	0.0348 (4)	
O11A	0.22078 (11)	0.58074 (10)	0.77936 (8)	0.0257 (3)	
O12A	−0.13915 (10)	0.19373 (10)	0.83970 (9)	0.0263 (3)	
O16A	0.50084 (11)	0.34114 (10)	0.38966 (8)	0.0278 (3)	
N1A	0.08853 (11)	0.63526 (11)	0.66747 (9)	0.0179 (3)	
C2A	−0.03768 (13)	0.64345 (13)	0.65383 (10)	0.0173 (4)	
C3A	−0.05318 (13)	0.69301 (13)	0.54299 (10)	0.0190 (4)	
C4A	−0.01771 (14)	0.80912 (14)	0.49119 (11)	0.0225 (4)	
C5A	0.09180 (15)	0.81439 (13)	0.52509 (11)	0.0234 (4)	
C6A	0.17338 (14)	0.69007 (13)	0.58437 (10)	0.0198 (4)	
C11A	0.11909 (14)	0.59241 (13)	0.75955 (11)	0.0200 (4)	
C12A	−0.04601 (17)	0.08158 (15)	0.83098 (14)	0.0340 (5)	
C16A	0.50805 (16)	0.36516 (16)	0.28637 (12)	0.0299 (5)	
C21A	−0.05983 (13)	0.52092 (13)	0.70245 (10)	0.0168 (4)	
C22A	0.01942 (13)	0.41768 (13)	0.67499 (10)	0.0188 (4)	
C23A	−0.00377 (13)	0.30662 (13)	0.71881 (11)	0.0191 (4)	
C24A	−0.10739 (13)	0.29805 (13)	0.79199 (11)	0.0187 (4)	
C25A	−0.18706 (13)	0.40017 (14)	0.82045 (10)	0.0200 (4)	
C26A	−0.16288 (13)	0.50999 (13)	0.77538 (10)	0.0187 (4)	
C31A	−0.18659 (15)	0.70981 (16)	0.52986 (12)	0.0267 (4)	
C61A	0.25694 (13)	0.60156 (13)	0.52662 (11)	0.0194 (4)	
C62A	0.26778 (13)	0.62969 (14)	0.42518 (11)	0.0213 (4)	
C63A	0.34838 (14)	0.54553 (14)	0.37608 (11)	0.0224 (4)	
C64A	0.41988 (13)	0.43110 (14)	0.42926 (11)	0.0212 (4)	
C65A	0.41148 (14)	0.40143 (14)	0.53153 (11)	0.0233 (4)	
C66A	0.33148 (14)	0.48552 (14)	0.57865 (11)	0.0219 (4)	
O4B	0.53820 (11)	0.36112 (12)	−0.07942 (10)	0.0332 (4)	
O11B	−0.01487 (10)	0.36764 (10)	0.00291 (8)	0.0257 (3)	
O12B	0.27346 (11)	−0.14716 (10)	−0.18039 (8)	0.0274 (3)	
O16B	0.30583 (13)	−0.04338 (11)	0.39872 (9)	0.0349 (4)	
N1B	0.20061 (11)	0.30115 (10)	−0.04000 (8)	0.0165 (3)	
C2B	0.30548 (13)	0.25962 (12)	−0.11508 (10)	0.0162 (3)	
C3B	0.43640 (13)	0.23049 (13)	−0.08605 (10)	0.0179 (3)	
C4B	0.44154 (14)	0.33596 (13)	−0.06091 (10)	0.0193 (4)	
C5B	0.31987 (13)	0.40727 (12)	−0.01243 (10)	0.0183 (4)	
C6B	0.22404 (13)	0.33873 (12)	0.03542 (10)	0.0167 (4)	
C11B	0.07901 (14)	0.32711 (13)	−0.05147 (11)	0.0196 (4)	
C12B	0.24507 (19)	−0.24519 (15)	−0.09881 (13)	0.0332 (5)	
C16B	0.39847 (19)	−0.06051 (17)	0.45455 (13)	0.0389 (5)	
C21B	0.29772 (12)	0.14959 (12)	−0.13105 (10)	0.0167 (4)	
C22B	0.29169 (14)	0.04595 (13)	−0.05153 (10)	0.0195 (4)	
C23B	0.28509 (14)	−0.05628 (13)	−0.06458 (11)	0.0200 (4)	
C24B	0.28423 (13)	−0.05435 (13)	−0.15938 (11)	0.0194 (4)	
C25B	0.29209 (13)	0.04776 (13)	−0.23975 (11)	0.0200 (4)	
C26B	0.29897 (13)	0.14859 (13)	−0.22514 (10)	0.0180 (4)	
C31B	0.54440 (14)	0.19367 (15)	−0.16552 (12)	0.0255 (4)	
C61B	0.25660 (13)	0.23214 (12)	0.12884 (10)	0.0173 (4)	
C62B	0.36078 (13)	0.20273 (13)	0.17407 (11)	0.0196 (4)	
C63B	0.38102 (14)	0.11155 (14)	0.26463 (11)	0.0228 (4)	
C64B	0.29614 (15)	0.04691 (13)	0.31014 (11)	0.0235 (4)	
C65B	0.19218 (15)	0.07336 (14)	0.26483 (11)	0.0252 (4)	
C66B	0.17247 (14)	0.16488 (14)	0.17595 (11)	0.0215 (4)	
H2A	−0.10363	0.70256	0.68675	0.0207*	
H3A	0.00755	0.62976	0.51192	0.0228*	
H5A	0.05791	0.86971	0.56616	0.0280*	
H5B	0.14629	0.84977	0.46661	0.0280*	
H6A	0.23149	0.70603	0.61370	0.0238*	
H11A	0.0512 (17)	0.5705 (16)	0.8096 (13)	0.019 (4)*	
H12A	−0.08033	0.01438	0.86816	0.0509*	
H12B	0.02979	0.06785	0.85741	0.0509*	
H12C	−0.02341	0.08532	0.76138	0.0509*	
H16A	0.56875	0.29407	0.26729	0.0448*	
H16B	0.53620	0.43645	0.25003	0.0448*	
H16C	0.42414	0.38148	0.27085	0.0448*	
H22A	0.09048	0.42328	0.62550	0.0226*	
H23A	0.05059	0.23725	0.69899	0.0230*	
H25A	−0.25758	0.39450	0.87049	0.0240*	
H26A	−0.21796	0.57951	0.79471	0.0225*	
H31A	−0.20497	0.63294	0.56433	0.0400*	
H31B	−0.19198	0.73357	0.45952	0.0400*	
H31C	−0.24850	0.77371	0.55730	0.0400*	
H62A	0.21928	0.70800	0.38819	0.0256*	
H63A	0.35409	0.56669	0.30660	0.0268*	
H65A	0.46083	0.32348	0.56837	0.0279*	
H66A	0.32664	0.46447	0.64805	0.0263*	
H2B	0.29644	0.32758	−0.17882	0.0194*	
H3B	0.44698	0.15857	−0.02494	0.0214*	
H5C	0.28080	0.48441	−0.06244	0.0220*	
H5D	0.33958	0.42867	0.03853	0.0220*	
H6B	0.14241	0.39896	0.05427	0.0200*	
H11B	0.0730 (16)	0.3100 (15)	−0.1086 (13)	0.014 (4)*	
H12D	0.23993	−0.30565	−0.12343	0.0498*	
H12E	0.16364	−0.21310	−0.06062	0.0498*	
H12F	0.31222	−0.28385	−0.05658	0.0498*	
H16D	0.39581	−0.12764	0.51595	0.0584*	
H16E	0.38045	0.01458	0.46998	0.0584*	
H16F	0.48296	−0.08026	0.41616	0.0584*	
H22B	0.29208	0.04497	0.01296	0.0233*	
H23B	0.28123	−0.12627	−0.00966	0.0240*	
H25B	0.29276	0.04850	−0.30439	0.0240*	
H26B	0.30460	0.21791	−0.28024	0.0217*	
H31D	0.53735	0.12570	−0.17918	0.0382*	
H31E	0.62606	0.16834	−0.14247	0.0382*	
H31F	0.53907	0.26335	−0.22589	0.0382*	
H62B	0.42003	0.24574	0.14265	0.0234*	
H63B	0.45232	0.09400	0.29474	0.0273*	
H65B	0.13468	0.02827	0.29520	0.0302*	
H66B	0.10079	0.18260	0.14623	0.0258*	

### Atomic displacement parameters (Å^2^)

**Table:**

	*U*^11^	*U*^22^	*U*^33^	*U*^12^	*U*^13^	*U*^23^
O4A	0.0407 (7)	0.0275 (6)	0.0286 (6)	−0.0091 (5)	−0.0121 (5)	0.0025 (5)
O11A	0.0285 (6)	0.0313 (6)	0.0210 (5)	−0.0145 (5)	−0.0062 (4)	−0.0053 (4)
O12A	0.0195 (5)	0.0203 (5)	0.0354 (6)	−0.0107 (4)	0.0024 (4)	−0.0044 (4)
O16A	0.0277 (6)	0.0263 (6)	0.0245 (6)	−0.0058 (5)	−0.0009 (4)	−0.0074 (4)
N1A	0.0186 (6)	0.0197 (6)	0.0160 (6)	−0.0105 (5)	0.0000 (4)	−0.0034 (4)
C2A	0.0166 (6)	0.0183 (7)	0.0162 (6)	−0.0068 (5)	0.0006 (5)	−0.0053 (5)
C3A	0.0176 (7)	0.0213 (7)	0.0162 (7)	−0.0060 (5)	−0.0003 (5)	−0.0056 (5)
C4A	0.0240 (7)	0.0211 (7)	0.0173 (7)	−0.0055 (6)	0.0011 (5)	−0.0051 (6)
C5A	0.0269 (8)	0.0188 (7)	0.0222 (7)	−0.0111 (6)	0.0001 (6)	−0.0025 (6)
C6A	0.0205 (7)	0.0221 (7)	0.0181 (7)	−0.0131 (6)	−0.0006 (5)	−0.0024 (5)
C11A	0.0235 (7)	0.0191 (7)	0.0183 (7)	−0.0101 (6)	−0.0009 (6)	−0.0048 (5)
C12A	0.0325 (9)	0.0210 (8)	0.0438 (10)	−0.0131 (7)	0.0074 (7)	−0.0090 (7)
C16A	0.0247 (8)	0.0368 (9)	0.0269 (8)	−0.0067 (7)	−0.0006 (6)	−0.0139 (7)
C21A	0.0168 (6)	0.0189 (7)	0.0160 (6)	−0.0080 (5)	−0.0019 (5)	−0.0048 (5)
C22A	0.0162 (6)	0.0220 (7)	0.0180 (7)	−0.0083 (5)	0.0024 (5)	−0.0069 (5)
C23A	0.0181 (7)	0.0188 (7)	0.0212 (7)	−0.0062 (5)	−0.0019 (5)	−0.0072 (5)
C24A	0.0172 (7)	0.0200 (7)	0.0206 (7)	−0.0095 (5)	−0.0045 (5)	−0.0032 (5)
C25A	0.0155 (6)	0.0254 (7)	0.0181 (7)	−0.0091 (5)	0.0016 (5)	−0.0057 (6)
C26A	0.0163 (6)	0.0216 (7)	0.0186 (7)	−0.0065 (5)	0.0001 (5)	−0.0079 (5)
C31A	0.0204 (7)	0.0344 (8)	0.0227 (7)	−0.0077 (6)	−0.0033 (6)	−0.0070 (6)
C61A	0.0161 (6)	0.0225 (7)	0.0196 (7)	−0.0115 (5)	−0.0001 (5)	−0.0028 (5)
C62A	0.0168 (7)	0.0241 (7)	0.0201 (7)	−0.0081 (6)	−0.0027 (5)	−0.0021 (6)
C63A	0.0191 (7)	0.0299 (8)	0.0176 (7)	−0.0114 (6)	−0.0020 (5)	−0.0036 (6)
C64A	0.0160 (7)	0.0249 (7)	0.0236 (7)	−0.0106 (6)	0.0012 (5)	−0.0071 (6)
C65A	0.0207 (7)	0.0223 (7)	0.0227 (7)	−0.0088 (6)	−0.0035 (6)	−0.0003 (6)
C66A	0.0213 (7)	0.0263 (7)	0.0169 (7)	−0.0122 (6)	−0.0011 (5)	−0.0017 (6)
O4B	0.0254 (6)	0.0402 (7)	0.0431 (7)	−0.0214 (5)	0.0049 (5)	−0.0178 (6)
O11B	0.0164 (5)	0.0288 (6)	0.0305 (6)	−0.0063 (4)	0.0005 (4)	−0.0113 (5)
O12B	0.0398 (7)	0.0208 (5)	0.0265 (6)	−0.0145 (5)	−0.0011 (5)	−0.0099 (4)
O16B	0.0435 (7)	0.0303 (6)	0.0246 (6)	−0.0189 (5)	−0.0075 (5)	0.0072 (5)
N1B	0.0163 (6)	0.0160 (5)	0.0174 (5)	−0.0061 (4)	−0.0008 (4)	−0.0055 (4)
C2B	0.0162 (6)	0.0148 (6)	0.0159 (6)	−0.0059 (5)	−0.0001 (5)	−0.0033 (5)
C3B	0.0158 (6)	0.0182 (6)	0.0180 (6)	−0.0068 (5)	0.0002 (5)	−0.0042 (5)
C4B	0.0206 (7)	0.0200 (7)	0.0172 (6)	−0.0109 (5)	−0.0016 (5)	−0.0019 (5)
C5B	0.0224 (7)	0.0144 (6)	0.0188 (7)	−0.0087 (5)	−0.0032 (5)	−0.0027 (5)
C6B	0.0162 (6)	0.0158 (6)	0.0186 (7)	−0.0059 (5)	−0.0003 (5)	−0.0066 (5)
C11B	0.0185 (7)	0.0176 (7)	0.0221 (7)	−0.0069 (5)	−0.0027 (5)	−0.0044 (5)
C12B	0.0491 (10)	0.0246 (8)	0.0339 (9)	−0.0223 (8)	−0.0018 (8)	−0.0096 (7)
C16B	0.0447 (10)	0.0321 (9)	0.0283 (9)	−0.0106 (8)	−0.0131 (8)	0.0067 (7)
C21B	0.0137 (6)	0.0148 (6)	0.0198 (7)	−0.0046 (5)	−0.0004 (5)	−0.0047 (5)
C22B	0.0219 (7)	0.0185 (7)	0.0168 (7)	−0.0074 (5)	−0.0009 (5)	−0.0045 (5)
C23B	0.0213 (7)	0.0153 (6)	0.0207 (7)	−0.0076 (5)	−0.0003 (5)	−0.0027 (5)
C24B	0.0162 (6)	0.0167 (6)	0.0254 (7)	−0.0050 (5)	0.0007 (5)	−0.0095 (6)
C25B	0.0196 (7)	0.0209 (7)	0.0187 (7)	−0.0056 (5)	−0.0007 (5)	−0.0076 (5)
C26B	0.0161 (6)	0.0167 (6)	0.0178 (7)	−0.0054 (5)	0.0002 (5)	−0.0032 (5)
C31B	0.0187 (7)	0.0324 (8)	0.0266 (8)	−0.0098 (6)	0.0034 (6)	−0.0132 (7)
C61B	0.0191 (7)	0.0150 (6)	0.0171 (7)	−0.0061 (5)	0.0017 (5)	−0.0063 (5)
C62B	0.0174 (7)	0.0179 (7)	0.0208 (7)	−0.0071 (5)	0.0025 (5)	−0.0052 (5)
C63B	0.0195 (7)	0.0217 (7)	0.0229 (7)	−0.0044 (6)	−0.0030 (6)	−0.0046 (6)
C64B	0.0289 (8)	0.0179 (7)	0.0187 (7)	−0.0082 (6)	0.0007 (6)	−0.0022 (6)
C65B	0.0301 (8)	0.0243 (7)	0.0229 (7)	−0.0174 (6)	0.0022 (6)	−0.0042 (6)
C66B	0.0233 (7)	0.0237 (7)	0.0207 (7)	−0.0118 (6)	−0.0004 (6)	−0.0077 (6)

### Geometric parameters (Å, °)

**Table:**

O4A—C4A	1.212 (2)	C25A—H25A	0.9500
O11A—C11A	1.225 (2)	C26A—H26A	0.9500
O12A—C12A	1.424 (2)	C31A—H31C	0.9800
O12A—C24A	1.369 (2)	C31A—H31B	0.9800
O16A—C16A	1.427 (2)	C31A—H31A	0.9800
O16A—C64A	1.368 (2)	C62A—H62A	0.9500
O4B—C4B	1.211 (2)	C63A—H63A	0.9500
O11B—C11B	1.224 (2)	C65A—H65A	0.9500
O12B—C24B	1.364 (2)	C66A—H66A	0.9500
O12B—C12B	1.433 (2)	C2B—C3B	1.548 (2)
O16B—C16B	1.423 (3)	C2B—C21B	1.519 (2)
O16B—C64B	1.366 (2)	C3B—C4B	1.523 (2)
N1A—C2A	1.482 (2)	C3B—C31B	1.528 (2)
N1A—C6A	1.480 (2)	C4B—C5B	1.502 (2)
N1A—C11A	1.349 (2)	C5B—C6B	1.525 (2)
N1B—C2B	1.4843 (19)	C6B—C61B	1.524 (2)
N1B—C6B	1.4794 (19)	C21B—C22B	1.394 (2)
N1B—C11B	1.354 (2)	C21B—C26B	1.392 (2)
C2A—C21A	1.518 (2)	C22B—C23B	1.394 (2)
C2A—C3A	1.5505 (19)	C23B—C24B	1.395 (2)
C3A—C4A	1.523 (2)	C24B—C25B	1.393 (2)
C3A—C31A	1.526 (3)	C25B—C26B	1.390 (2)
C4A—C5A	1.509 (3)	C61B—C62B	1.387 (2)
C5A—C6A	1.530 (2)	C61B—C66B	1.404 (2)
C6A—C61A	1.527 (2)	C62B—C63B	1.398 (2)
C21A—C22A	1.395 (2)	C63B—C64B	1.387 (2)
C21A—C26A	1.391 (2)	C64B—C65B	1.395 (3)
C22A—C23A	1.392 (2)	C65B—C66B	1.382 (2)
C23A—C24A	1.394 (2)	C2B—H2B	1.0000
C24A—C25A	1.393 (2)	C3B—H3B	1.0000
C25A—C26A	1.384 (2)	C5B—H5C	0.9900
C61A—C62A	1.388 (2)	C5B—H5D	0.9900
C61A—C66A	1.405 (2)	C6B—H6B	1.0000
C62A—C63A	1.399 (2)	C11B—H11B	0.971 (18)
C63A—C64A	1.386 (2)	C12B—H12D	0.9800
C64A—C65A	1.400 (2)	C12B—H12E	0.9800
C65A—C66A	1.378 (2)	C12B—H12F	0.9800
C2A—H2A	1.0000	C16B—H16D	0.9800
C3A—H3A	1.0000	C16B—H16E	0.9800
C5A—H5B	0.9900	C16B—H16F	0.9800
C5A—H5A	0.9900	C22B—H22B	0.9500
C6A—H6A	1.0000	C23B—H23B	0.9500
C11A—H11A	0.97 (2)	C25B—H25B	0.9500
C12A—H12B	0.9800	C26B—H26B	0.9500
C12A—H12A	0.9800	C31B—H31D	0.9800
C12A—H12C	0.9800	C31B—H31E	0.9800
C16A—H16B	0.9800	C31B—H31F	0.9800
C16A—H16C	0.9800	C62B—H62B	0.9500
C16A—H16A	0.9800	C63B—H63B	0.9500
C22A—H22A	0.9500	C65B—H65B	0.9500
C23A—H23A	0.9500	C66B—H66B	0.9500
			
O4B···C25A^i^	3.266 (2)	C66B···H2A^vii^	2.8600
O11A···C5B^ii^	3.2221 (18)	H2A···H11A	2.5200
O11A···C66A	3.3659 (19)	H2A···H26A	2.3000
O11A···C16A^iii^	3.278 (2)	H2A···C64B^vii^	3.0600
O11B···C12B^iv^	3.309 (3)	H2A···C65B^vii^	2.8800
O11B···C66B	3.378 (2)	H2A···C66B^vii^	2.8600
O16A···C16B^v^	3.334 (2)	H2A···C61B^vii^	3.0400
O16B···C5A^vi^	3.351 (2)	H2A···H31C	2.5500
O4A···H31C	2.8500	H2B···H26B	2.3500
O4A···H12C^vii^	2.6500	H2B···H11B	2.5800
O4A···H31B	2.6600	H2B···O11A^xv^	2.8000
O4A···H23A^vii^	2.7900	H2B···C5B	3.0900
O4B···H23B^viii^	2.9100	H2B···C11A^xv^	3.0700
O4B···H31E	2.6400	H2B···H31F	2.5700
O4B···H31F	2.8600	H2B···H66A^xv^	2.5100
O4B···H16B^ix^	2.8500	H3A···C61A	2.8200
O4B···H12F^viii^	2.6500	H3A···C6A	2.8600
O4B···H25A^i^	2.4300	H3A···C22A	2.8200
O11A···H5C^ii^	2.3400	H3A···H22A	2.4600
O11A···H6A	2.3600	H3A···C62A	2.9400
O11A···H66A	2.6200	H3B···C22B	2.8500
O11A···H16B^iii^	2.6600	H3B···C6B	2.8800
O11A···H2B^ii^	2.8000	H3B···H22B	2.5200
O11A···H12D^x^	2.4700	H3B···C61B	2.8700
O11B···H12E^iv^	2.8200	H3B···C62B	3.0300
O11B···H6B^xi^	2.6200	H5B···O16B^xiv^	2.4700
O11B···H66B	2.6500	H5B···C62A	2.7800
O11B···H6B	2.3500	H5B···H62A	2.2500
O12A···H31E^xii^	2.7800	H5C···C2B	3.0800
O12A···H23B^xiii^	2.6500	H5C···C12B^xiv^	3.0900
O12B···H16A^viii^	2.5100	H5C···O11A^xv^	2.3400
O16A···H16D^v^	2.4700	H5C···H12D^xiv^	2.3200
O16A···H6A^iii^	2.9000	H5D···H26A^vii^	2.4800
O16B···H5B^vi^	2.4700	H5D···H62B	2.2200
N1A···H22A	2.9300	H5D···C62B	2.7400
N1A···H66A	2.7700	H6A···O16A^iii^	2.9000
N1B···H66B	2.7700	H6A···O11A	2.3600
N1B···H22B	2.8100	H6B···O11B	2.3500
C3A···C61A	3.278 (2)	H6B···O11B^xi^	2.6200
C3A···C62A	3.569 (2)	H6B···H11A^vii^	2.5900
C3B···C61B	3.307 (2)	H11A···C11B^ii^	2.908 (19)
C4A···C62A	3.323 (2)	H11A···C21A	2.64 (2)
C4B···C62B	3.294 (2)	H11A···C26A	3.06 (2)
C5A···O16B^xiv^	3.351 (2)	H11A···H2A	2.5200
C5B···O11A^xv^	3.2221 (18)	H11A···H6B^vii^	2.5900
C11A···C66A	3.411 (2)	H11B···H2B	2.5800
C11A···C11B^ii^	3.542 (2)	H11B···C25A^xv^	3.09 (2)
C11A···C22A	3.524 (2)	H11B···C22A^xv^	3.095 (18)
C11B···C66B	3.414 (2)	H11B···C23A^xv^	2.932 (19)
C11B···C11A^xv^	3.542 (2)	H11B···C21B	2.619 (19)
C11B···C22B	3.416 (2)	H11B···C24A^xv^	2.92 (2)
C12A···C24B^ii^	3.536 (3)	H12B···C24B^ii^	2.7300
C12B···O11B^iv^	3.309 (3)	H12B···C25B^ii^	2.9500
C16A···O11A^iii^	3.278 (2)	H12B···C23B^ii^	3.0500
C16B···O16A^v^	3.334 (2)	H12B···C23A	2.8600
C22A···C11A	3.524 (2)	H12B···H23A	2.5300
C22B···C11B	3.416 (2)	H12C···H23A	2.1700
C23A···C26B^ii^	3.424 (2)	H12C···C23A	2.6800
C24B···C12A^xv^	3.536 (3)	H12C···O4A^vii^	2.6500
C25A···O4B^xii^	3.266 (2)	H12D···O11A^xvi^	2.4700
C25A···C63A^vii^	3.571 (2)	H12D···H5C^vi^	2.3200
C25B···C63B^viii^	3.509 (2)	H12E···C23B	2.8000
C26A···C31A	3.556 (2)	H12E···H23B	2.4300
C26B···C31B	3.471 (2)	H12E···O11B^iv^	2.8200
C26B···C23A^xv^	3.424 (2)	H12F···H23B	2.2000
C31A···C26A	3.556 (2)	H12F···O4B^viii^	2.6500
C31A···C64A^vii^	3.570 (3)	H12F···C23B	2.7000
C31B···C26B	3.471 (2)	H16A···O12B^viii^	2.5100
C61A···C3A	3.278 (2)	H16A···C12B^viii^	2.9100
C61B···C3B	3.307 (2)	H16B···C63A	2.7400
C62A···C4A	3.323 (2)	H16B···O11A^iii^	2.6600
C62A···C3A	3.569 (2)	H16B···H63A	2.2800
C62B···C4B	3.294 (2)	H16B···O4B^ix^	2.8500
C63A···C25A^vii^	3.571 (2)	H16C···C26A^vii^	2.9300
C63B···C25B^viii^	3.509 (2)	H16C···C63A	2.7700
C64A···C66A^iii^	3.375 (2)	H16C···H63A	2.3500
C64A···C31A^vii^	3.570 (3)	H16D···O16A^v^	2.4700
C65A···C66A^iii^	3.525 (2)	H16E···H63B	2.4400
C65A···C65A^iii^	3.506 (2)	H16E···H31C^vii^	2.4200
C66A···C64A^iii^	3.375 (2)	H16E···C63B	2.8100
C66A···O11A	3.3659 (19)	H16F···H63B	2.2100
C66A···C11A	3.411 (2)	H16F···C63B	2.7000
C66A···C65A^iii^	3.525 (2)	H22A···C3A	3.0400
C66B···O11B	3.378 (2)	H22A···N1A	2.9300
C66B···C11B	3.414 (2)	H22A···H3A	2.4600
C2B···H5C	3.0800	H22A···C31A^vii^	3.0600
C3A···H22A	3.0400	H22A···C66A	3.0100
C3B···H22B	3.0700	H22A···H31B^vii^	2.4700
C4A···H62A	2.8200	H22B···N1B	2.8100
C4B···H62B	2.7700	H22B···C3B	3.0700
C5A···H62A	2.6400	H22B···C66B	3.0500
C5B···H2B	3.0900	H22B···H3B	2.5200
C5B···H62B	2.6000	H23A···C26B^ii^	3.0000
C6A···H3A	2.8600	H23A···C12A	2.5500
C6B···H26A^vii^	3.0200	H23A···C25B^ii^	2.9600
C6B···H3B	2.8800	H23A···H12B	2.5300
C11A···H66A	2.8200	H23A···H12C	2.1700
C11A···H2B^ii^	3.0700	H23A···O4A^vii^	2.7900
C11B···H66B	2.8100	H23A···H31B^vii^	2.4700
C11B···H11A^xv^	2.908 (19)	H23B···O12A^xiii^	2.6500
C12A···H23A	2.5500	H23B···H31E^viii^	2.5300
C12B···H16A^viii^	2.9100	H23B···H12F	2.2000
C12B···H5C^vi^	3.0900	H23B···C12B	2.5200
C12B···H23B	2.5200	H23B···H12E	2.4300
C16A···H63A	2.5300	H23B···O4B^viii^	2.9100
C16B···H63B	2.5300	H25A···O4B^xii^	2.4300
C21A···H31A	2.6000	H26A···C6B^vii^	3.0200
C21A···H11A	2.64 (2)	H26A···H2A	2.3000
C21B···H31D	2.5900	H26A···H5D^vii^	2.4800
C21B···H11B	2.619 (19)	H26A···C61B^vii^	2.8200
C22A···H3A	2.8200	H26A···C62B^vii^	2.7200
C22A···H31B^vii^	3.0500	H26B···H2B	2.3500
C22A···H11B^ii^	3.095 (18)	H31A···C21A	2.6000
C22B···H3B	2.8500	H31A···C26A	2.9900
C23A···H12B	2.8600	H31A···C63A^vii^	3.0200
C23A···H11B^ii^	2.932 (19)	H31A···C64A^vii^	2.8600
C23A···H31B^vii^	3.0500	H31B···O4A	2.6600
C23A···H12C	2.6800	H31B···C22A^vii^	3.0500
C23B···H12B^xv^	3.0500	H31B···C23A^vii^	3.0500
C23B···H12E	2.8000	H31B···H22A^vii^	2.4700
C23B···H12F	2.7000	H31B···H23A^vii^	2.4700
C24A···H11B^ii^	2.92 (2)	H31C···O4A	2.8500
C24B···H12B^xv^	2.7300	H31C···H2A	2.5500
C25A···H11B^ii^	3.09 (2)	H31C···H16E^vii^	2.4200
C25A···H63A^vii^	2.8500	H31D···C21B	2.5900
C25B···H23A^xv^	2.9600	H31D···C26B	2.8900
C25B···H63B^viii^	2.8700	H31E···H23B^viii^	2.5300
C25B···H12B^xv^	2.9500	H31E···O4B	2.6400
C26A···H16C^vii^	2.9300	H31E···O12A^i^	2.7800
C26A···H31A	2.9900	H31F···H2B	2.5700
C26A···H11A	3.06 (2)	H31F···C63A^ix^	3.0400
C26B···H23A^xv^	3.0000	H31F···O4B	2.8600
C26B···H31D	2.8900	H62A···C4A	2.8200
C31A···H22A^vii^	3.0600	H62A···C5A	2.6400
C61A···H3A	2.8200	H62A···H5B	2.2500
C61B···H26A^vii^	2.8200	H62B···C4B	2.7700
C61B···H3B	2.8700	H62B···C5B	2.6000
C61B···H2A^vii^	3.0400	H62B···H5D	2.2200
C62A···H5B	2.7800	H63A···C25A^vii^	2.8500
C62A···H3A	2.9400	H63A···C16A	2.5300
C62B···H5D	2.7400	H63A···H16B	2.2800
C62B···H26A^vii^	2.7200	H63A···H16C	2.3500
C62B···H3B	3.0300	H63B···H16E	2.4400
C63A···H16C	2.7700	H63B···H16F	2.2100
C63A···H16B	2.7400	H63B···C25B^viii^	2.8700
C63A···H31A^vii^	3.0200	H63B···C16B	2.5300
C63A···H31F^ix^	3.0400	H66A···O11A	2.6200
C63B···H16F	2.7000	H66A···N1A	2.7700
C63B···H16E	2.8100	H66A···C11A	2.8200
C64A···H31A^vii^	2.8600	H66A···H2B^ii^	2.5100
C64B···H2A^vii^	3.0600	H66B···O11B	2.6500
C65B···H2A^vii^	2.8800	H66B···N1B	2.7700
C66A···H22A	3.0100	H66B···C11B	2.8100
C66B···H22B	3.0500		
			
C12A—O12A—C24A	117.51 (14)	C64A—C63A—H63A	120.00
C16A—O16A—C64A	117.41 (13)	C66A—C65A—H65A	120.00
C12B—O12B—C24B	117.35 (12)	C64A—C65A—H65A	120.00
C16B—O16B—C64B	117.60 (15)	C65A—C66A—H66A	119.00
C2A—N1A—C11A	119.14 (13)	C61A—C66A—H66A	119.00
C2A—N1A—C6A	121.23 (12)	N1B—C2B—C3B	111.14 (12)
C6A—N1A—C11A	118.97 (14)	N1B—C2B—C21B	111.06 (12)
C2B—N1B—C6B	120.82 (13)	C3B—C2B—C21B	110.55 (12)
C2B—N1B—C11B	119.48 (12)	C2B—C3B—C4B	111.18 (13)
C6B—N1B—C11B	118.52 (12)	C2B—C3B—C31B	111.32 (12)
N1A—C2A—C3A	111.04 (12)	C4B—C3B—C31B	112.34 (14)
N1A—C2A—C21A	111.08 (13)	O4B—C4B—C3B	121.78 (15)
C3A—C2A—C21A	111.07 (12)	O4B—C4B—C5B	121.96 (15)
C2A—C3A—C31A	110.73 (13)	C3B—C4B—C5B	116.27 (14)
C4A—C3A—C31A	112.49 (14)	C4B—C5B—C6B	113.58 (12)
C2A—C3A—C4A	111.44 (13)	N1B—C6B—C5B	108.45 (11)
O4A—C4A—C3A	122.36 (16)	N1B—C6B—C61B	111.82 (12)
O4A—C4A—C5A	121.66 (16)	C5B—C6B—C61B	115.96 (13)
C3A—C4A—C5A	115.98 (13)	O11B—C11B—N1B	125.09 (15)
C4A—C5A—C6A	113.82 (14)	C2B—C21B—C22B	120.51 (12)
C5A—C6A—C61A	116.35 (12)	C2B—C21B—C26B	121.05 (13)
N1A—C6A—C61A	111.55 (13)	C22B—C21B—C26B	118.42 (14)
N1A—C6A—C5A	107.83 (13)	C21B—C22B—C23B	121.46 (13)
O11A—C11A—N1A	124.69 (15)	C22B—C23B—C24B	119.09 (14)
C2A—C21A—C26A	119.91 (14)	O12B—C24B—C23B	124.13 (14)
C22A—C21A—C26A	118.19 (14)	O12B—C24B—C25B	115.70 (13)
C2A—C21A—C22A	121.89 (13)	C23B—C24B—C25B	120.16 (15)
C21A—C22A—C23A	121.19 (14)	C24B—C25B—C26B	119.82 (14)
C22A—C23A—C24A	119.45 (14)	C21B—C26B—C25B	121.04 (13)
O12A—C24A—C25A	115.65 (14)	C6B—C61B—C62B	123.48 (14)
O12A—C24A—C23A	124.33 (14)	C6B—C61B—C66B	118.65 (14)
C23A—C24A—C25A	120.03 (14)	C62B—C61B—C66B	117.72 (13)
C24A—C25A—C26A	119.60 (14)	C61B—C62B—C63B	121.82 (15)
C21A—C26A—C25A	121.54 (14)	C62B—C63B—C64B	119.44 (16)
C6A—C61A—C66A	118.14 (13)	O16B—C64B—C63B	124.57 (16)
C62A—C61A—C66A	117.57 (14)	O16B—C64B—C65B	115.81 (16)
C6A—C61A—C62A	124.25 (14)	C63B—C64B—C65B	119.63 (14)
C61A—C62A—C63A	121.60 (15)	C64B—C65B—C66B	120.28 (16)
C62A—C63A—C64A	119.71 (14)	C61B—C66B—C65B	121.11 (16)
O16A—C64A—C65A	115.42 (14)	N1B—C2B—H2B	108.00
O16A—C64A—C63A	124.99 (14)	C3B—C2B—H2B	108.00
C63A—C64A—C65A	119.59 (15)	C21B—C2B—H2B	108.00
C64A—C65A—C66A	119.88 (15)	C2B—C3B—H3B	107.00
C61A—C66A—C65A	121.64 (14)	C4B—C3B—H3B	107.00
C21A—C2A—H2A	108.00	C31B—C3B—H3B	107.00
N1A—C2A—H2A	108.00	C4B—C5B—H5C	109.00
C3A—C2A—H2A	108.00	C4B—C5B—H5D	109.00
C2A—C3A—H3A	107.00	C6B—C5B—H5C	109.00
C4A—C3A—H3A	107.00	C6B—C5B—H5D	109.00
C31A—C3A—H3A	107.00	H5C—C5B—H5D	108.00
C4A—C5A—H5A	109.00	N1B—C6B—H6B	107.00
C6A—C5A—H5B	109.00	C5B—C6B—H6B	107.00
H5A—C5A—H5B	108.00	C61B—C6B—H6B	107.00
C4A—C5A—H5B	109.00	O11B—C11B—H11B	122.3 (12)
C6A—C5A—H5A	109.00	N1B—C11B—H11B	112.6 (12)
N1A—C6A—H6A	107.00	O12B—C12B—H12D	109.00
C61A—C6A—H6A	107.00	O12B—C12B—H12E	109.00
C5A—C6A—H6A	107.00	O12B—C12B—H12F	109.00
O11A—C11A—H11A	122.9 (12)	H12D—C12B—H12E	109.00
N1A—C11A—H11A	112.5 (12)	H12D—C12B—H12F	109.00
O12A—C12A—H12B	109.00	H12E—C12B—H12F	109.00
O12A—C12A—H12C	109.00	O16B—C16B—H16D	109.00
O12A—C12A—H12A	109.00	O16B—C16B—H16E	109.00
H12B—C12A—H12C	109.00	O16B—C16B—H16F	109.00
H12A—C12A—H12B	109.00	H16D—C16B—H16E	109.00
H12A—C12A—H12C	109.00	H16D—C16B—H16F	109.00
O16A—C16A—H16C	109.00	H16E—C16B—H16F	109.00
H16A—C16A—H16B	109.00	C21B—C22B—H22B	119.00
H16A—C16A—H16C	109.00	C23B—C22B—H22B	119.00
H16B—C16A—H16C	109.00	C22B—C23B—H23B	120.00
O16A—C16A—H16A	109.00	C24B—C23B—H23B	120.00
O16A—C16A—H16B	109.00	C24B—C25B—H25B	120.00
C23A—C22A—H22A	119.00	C26B—C25B—H25B	120.00
C21A—C22A—H22A	119.00	C21B—C26B—H26B	119.00
C22A—C23A—H23A	120.00	C25B—C26B—H26B	119.00
C24A—C23A—H23A	120.00	C3B—C31B—H31D	109.00
C26A—C25A—H25A	120.00	C3B—C31B—H31E	109.00
C24A—C25A—H25A	120.00	C3B—C31B—H31F	109.00
C21A—C26A—H26A	119.00	H31D—C31B—H31E	109.00
C25A—C26A—H26A	119.00	H31D—C31B—H31F	109.00
H31B—C31A—H31C	109.00	H31E—C31B—H31F	109.00
C3A—C31A—H31B	109.00	C61B—C62B—H62B	119.00
H31A—C31A—H31C	109.00	C63B—C62B—H62B	119.00
C3A—C31A—H31A	109.00	C62B—C63B—H63B	120.00
C3A—C31A—H31C	109.00	C64B—C63B—H63B	120.00
H31A—C31A—H31B	109.00	C64B—C65B—H65B	120.00
C61A—C62A—H62A	119.00	C66B—C65B—H65B	120.00
C63A—C62A—H62A	119.00	C61B—C66B—H66B	119.00
C62A—C63A—H63A	120.00	C65B—C66B—H66B	119.00
			
C12A—O12A—C24A—C23A	15.1 (2)	C22A—C23A—C24A—C25A	0.3 (2)
C12A—O12A—C24A—C25A	−165.35 (15)	O12A—C24A—C25A—C26A	−179.35 (14)
C16A—O16A—C64A—C63A	−2.8 (3)	C23A—C24A—C25A—C26A	0.2 (2)
C16A—O16A—C64A—C65A	177.15 (16)	C24A—C25A—C26A—C21A	−0.5 (2)
C12B—O12B—C24B—C23B	8.3 (2)	C66A—C61A—C62A—C63A	0.9 (3)
C12B—O12B—C24B—C25B	−170.39 (16)	C6A—C61A—C62A—C63A	178.45 (16)
C16B—O16B—C64B—C65B	−170.52 (15)	C6A—C61A—C66A—C65A	−178.56 (16)
C16B—O16B—C64B—C63B	9.3 (2)	C62A—C61A—C66A—C65A	−0.9 (3)
C6A—N1A—C2A—C21A	134.29 (13)	C61A—C62A—C63A—C64A	−0.2 (3)
C11A—N1A—C2A—C3A	−179.22 (13)	C62A—C63A—C64A—O16A	179.39 (16)
C11A—N1A—C2A—C21A	−55.10 (18)	C62A—C63A—C64A—C65A	−0.6 (3)
C6A—N1A—C2A—C3A	10.16 (19)	C63A—C64A—C65A—C66A	0.6 (3)
C2A—N1A—C11A—O11A	179.34 (15)	O16A—C64A—C65A—C66A	−179.35 (16)
C6A—N1A—C11A—O11A	−9.8 (2)	C64A—C65A—C66A—C61A	0.1 (3)
C2A—N1A—C6A—C61A	−87.54 (17)	N1B—C2B—C3B—C4B	−51.16 (15)
C11A—N1A—C6A—C5A	−129.24 (15)	N1B—C2B—C3B—C31B	−177.23 (12)
C2A—N1A—C6A—C5A	41.39 (18)	C21B—C2B—C3B—C4B	−174.96 (11)
C11A—N1A—C6A—C61A	101.83 (16)	C21B—C2B—C3B—C31B	58.96 (16)
C11B—N1B—C2B—C3B	−177.91 (13)	N1B—C2B—C21B—C22B	−53.80 (19)
C11B—N1B—C2B—C21B	−54.40 (17)	N1B—C2B—C21B—C26B	127.48 (15)
C2B—N1B—C11B—O11B	−176.20 (14)	C3B—C2B—C21B—C22B	70.05 (18)
C6B—N1B—C11B—O11B	−8.5 (2)	C3B—C2B—C21B—C26B	−108.67 (16)
C11B—N1B—C6B—C5B	−129.69 (14)	C2B—C3B—C4B—O4B	−145.88 (15)
C2B—N1B—C6B—C5B	37.80 (17)	C2B—C3B—C4B—C5B	33.80 (16)
C2B—N1B—C6B—C61B	−91.30 (15)	C31B—C3B—C4B—O4B	−20.4 (2)
C6B—N1B—C2B—C21B	138.23 (13)	C31B—C3B—C4B—C5B	159.31 (13)
C11B—N1B—C6B—C61B	101.21 (15)	O4B—C4B—C5B—C6B	−160.48 (14)
C6B—N1B—C2B—C3B	14.71 (17)	C3B—C4B—C5B—C6B	19.84 (17)
N1A—C2A—C3A—C4A	−49.92 (17)	C4B—C5B—C6B—N1B	−55.45 (16)
C3A—C2A—C21A—C26A	−114.74 (16)	C4B—C5B—C6B—C61B	71.30 (16)
N1A—C2A—C3A—C31A	−175.94 (13)	N1B—C6B—C61B—C62B	130.09 (15)
C21A—C2A—C3A—C4A	−174.06 (13)	N1B—C6B—C61B—C66B	−54.52 (18)
C21A—C2A—C3A—C31A	59.92 (17)	C5B—C6B—C61B—C62B	5.1 (2)
N1A—C2A—C21A—C22A	−60.01 (18)	C5B—C6B—C61B—C66B	−179.56 (13)
N1A—C2A—C21A—C26A	121.16 (15)	C2B—C21B—C22B—C23B	−179.71 (15)
C3A—C2A—C21A—C22A	64.10 (19)	C26B—C21B—C22B—C23B	−1.0 (2)
C2A—C3A—C4A—O4A	−143.90 (16)	C2B—C21B—C26B—C25B	179.89 (15)
C31A—C3A—C4A—O4A	−18.9 (2)	C22B—C21B—C26B—C25B	1.1 (2)
C31A—C3A—C4A—C5A	161.50 (13)	C21B—C22B—C23B—C24B	−0.2 (3)
C2A—C3A—C4A—C5A	36.46 (19)	C22B—C23B—C24B—O12B	−177.55 (16)
C3A—C4A—C5A—C6A	16.50 (19)	C22B—C23B—C24B—C25B	1.1 (3)
O4A—C4A—C5A—C6A	−163.15 (15)	O12B—C24B—C25B—C26B	177.84 (15)
C4A—C5A—C6A—C61A	71.46 (19)	C23B—C24B—C25B—C26B	−0.9 (3)
C4A—C5A—C6A—N1A	−54.69 (16)	C24B—C25B—C26B—C21B	−0.2 (3)
N1A—C6A—C61A—C66A	−54.5 (2)	C6B—C61B—C62B—C63B	173.88 (14)
C5A—C6A—C61A—C62A	3.7 (2)	C66B—C61B—C62B—C63B	−1.6 (2)
N1A—C6A—C61A—C62A	127.93 (17)	C6B—C61B—C66B—C65B	−175.01 (14)
C5A—C6A—C61A—C66A	−178.79 (16)	C62B—C61B—C66B—C65B	0.6 (2)
C22A—C21A—C26A—C25A	0.3 (2)	C61B—C62B—C63B—C64B	1.1 (2)
C2A—C21A—C26A—C25A	179.15 (14)	C62B—C63B—C64B—O16B	−179.58 (15)
C2A—C21A—C22A—C23A	−178.60 (14)	C62B—C63B—C64B—C65B	0.3 (2)
C26A—C21A—C22A—C23A	0.3 (2)	O16B—C64B—C65B—C66B	178.71 (15)
C21A—C22A—C23A—C24A	−0.5 (2)	C63B—C64B—C65B—C66B	−1.1 (2)
C22A—C23A—C24A—O12A	179.82 (15)	C64B—C65B—C66B—C61B	0.7 (2)

Symmetry codes: (i) *x*+1, *y*, *z*−1; (ii) *x*, *y*, *z*+1; (iii) −*x*+1, −*y*+1, −*z*+1; (iv) −*x*, −*y*, −*z*; (v) −*x*+1, −*y*, −*z*+1; (vi) *x*, *y*−1, *z*; (vii) −*x*, −*y*+1, −*z*+1; (viii) −*x*+1, −*y*, −*z*; (ix) −*x*+1, −*y*+1, −*z*; (x) *x*, *y*+1, *z*+1; (xi) −*x*, −*y*+1, −*z*; (xii) *x*−1, *y*, *z*+1; (xiii) −*x*, −*y*, −*z*+1; (xiv) *x*, *y*+1, *z*; (xv) *x*, *y*, *z*−1; (xvi) *x*, *y*−1, *z*−1.

### Hydrogen-bond geometry (Å, °)

**Table:**

*D*—H···*A*	*D*—H	H···*A*	*D*···*A*	*D*—H···*A*
C5A—H5B···O16B^xiv^	0.99	2.47	3.351 (2)	148
C5B—H5C···O11A^xv^	0.99	2.34	3.222 (2)	148
C12B—H12D···O11A^xvi^	0.98	2.47	3.427 (2)	167
C16A—H16A···O12B^viii^	0.98	2.51	3.468 (2)	167
C16B—H16D···O16A^v^	0.98	2.47	3.334 (2)	147
C25A—H25A···O4B^xii^	0.95	2.43	3.266 (2)	147
C2A—H2A···Cg1^vii^	1.00	2.71	3.714 (2)	178
C12A—H12B···Cg2^ii^	0.98	2.92	3.846 (2)	158

Symmetry codes: (xiv) *x*, *y*+1, *z*; (xv) *x*, *y*, *z*−1; (xvi) *x*, *y*−1, *z*−1; (viii) −*x*+1, −*y*, −*z*; (v) −*x*+1, −*y*, −*z*+1; (xii) *x*−1, *y*, *z*+1; (vii) −*x*, −*y*+1, −*z*+1; (ii) *x*, *y*, *z*+1.
